# Neurocritical care physicians’ doubt about whether to withdraw life-sustaining treatment the first days after devastating brain injury: an interview study

**DOI:** 10.1186/s13049-019-0648-9

**Published:** 2019-08-28

**Authors:** Annette Robertsen, Eirik Helseth, Jon Henrik Laake, Reidun Førde

**Affiliations:** 1 Department of Anaesthesiology, Division of Emergencies and Critical Care, Oslo University Hospital, Oslo, Norway; 2 Department of Clinical Medicine, University of Oslo, Oslo, Norway; 3 Department of Neurosurgery, Oslo University Hospital, Oslo, Norway; 4 Centre of Medical Ethics, University of Oslo, Oslo, Norway

**Keywords:** Decision-making, Ethics, Severe traumatic brain injury, Devastating brain injury, Potentially inappropriate treatment

## Abstract

**Background:**

Multilevel uncertainty exists in the treatment of devastating brain injury and variation in end-of-life decision-making is a concern. Cognitive and emotional doubt linked to making challenging decisions have not received much attention. The aim of this study was to explore physicians´ doubt related to decisions to withhold or withdraw life-sustaining treatment within the first 72 h after devastating brain injury and to identify the strategies used to address doubt.

**Method:**

Semi-structured interviews were conducted with 18 neurocritical care physicians in a Norwegian trauma centre (neurosurgeons, intensivists and rehabilitation specialists) followed by a qualitative thematic analysis.

**Result:**

All physicians described feelings of doubt. The degree of doubt and how they dealt with it varied. Institutional culture, ethics climate and individual physicians´ values, experiences and emotions seemed to impact judgements and decisions. Common strategies applied by physicians across specialities when dealing with uncertainty and doubt were: 1. Provision of treatment trials 2. Using time as a coping strategy 3. Collegial counselling and interdisciplinary consensus seeking 4. Framing decisions as purely medical.

**Conclusion:**

Decisions regarding life-sustaining treatment after devastating brain injury are crafted in a stepwise manner. Feelings of doubt are frequent and seem to be linked to the recognition of fallibility. Doubt can be seen as positive and can foster open-mindedness towards the view of others, which is one of the prerequisites for a good ethical climate. Doubt in this context tends to be mitigated by open interdisciplinary discussions acknowledging doubt as rational and a normal feature of complex decision-making.

## Background

Decisions regarding whether to start, continue, limit or withdraw treatment in cases of severe traumatic brain injury (sTBI) are made on a regular basis in trauma hospitals [1–5]. Decisions can be ethically and emotionally challenging for all involved parts.

Devastating brain injury (DBI) is defined as an injury *assessed* as an immediate threat to life or incompatible with good functional recovery and where physicians *consider* early limitations or withdrawal of therapy *already at the time of hospital admission* [6, 7].

Cognitive and emotional doubt linked to many of these challenging decisions, have not received much scientific attention.

Bosslet et al. differentiate the *futile from the potentially inappropriate cases,* defining futility as “intended physiological goals are not possible to achieve” [8]. Potentially inappropriate treatment, on the other hand, is a concept pointing towards more complex decisions that include different medical and ethical considerations weighted against each other. Usually final conclusions are reached following a stepwise approach whereby it is realised that intermediate goals may be obtainable, but treatment may still not be in the individual patient’s best interest, e.g., when the life of the patient is saved but the neurological outcome is unacceptably bad [8–12]. The difference is ethically important. Patient’s values, wishes and will definitely should bear weight in the latter situation.

In sTBI, there is a lack of reliable prognostic tools early after injury [2]. If physicians are too pessimistic too early, self-fulfilling prophecies are a risk [6, 7, 13, 14].

Concern has been raised about variability between both individual clinicians and different institutions regarding end-of-life practices in sTBI [4, 15–17]. These variations may be seen as a threat to treatment quality and to the ideal of equality and justice for patients.

International guidelines for the management of devastating brain injury (DBI) have recently been developed in order to reduce practice variability [6, 7]. They recommend that physicians delay decisions regarding withdrawal of life-sustaining treatment for 48–72 h. Delays allow opportunities for prognostic evaluation, care planning and considerations of organ donation. Time-sensitive interventions (neurosurgery, treating intracranial hypertension) should be undertaken without delay when these are potentially meaningful [6, 7]. A shared decision model that requires attention to patients’ values and wishes is to be used [18].

The Oslo University Hospital brain injury guidelines, Norwegian national ethics guidelines and Norwegian law are all in accordance with these recommendations but without the provision of specific time frames. Additionally, in Norway, the final decision-making authority for patients without decision-making capacity lies with the physician in charge, even though the law requires a shared process prior to decisions [19, 20].

The aim of the study was to explore clinicians’ doubt related to dealing with DBI cases and the strategies they use when end-of-life decisions are made. We focus on the first 72 h after hospitalization in a Norwegian trauma hospital setting.

## Materials and methods

### Setting and study participants.

Oslo University Hospital (OUH) is the largest trauma centre in Norway, with a catchment area covering half the Norwegian population. In the OUH neurocritical care unit rehabilitation physicians are involved from a very early stage. Trauma patients are cared for using team-approaches. There are several mechanisms for providing feedback to individual clinicians; daily reviews of treatment decisions, meetings with weekly case reviews and mortality and morbidity conferences that address how to continuously improve system factors. For OUH local recommendations on TBI prognostication, communication and ethical matters, see Table 1.

**Table 1:** From the Oslo University Hospital local recommendation on sTBI care

Communication with the family	
Identify who is next of kin. Structured family meetings should involve conversations about diagnosis, treatment, prognosis and plan. Repeated updates on patient’s medical status and plans are needed. Strive for continuity of care. Briefings within the team, prior to family meetings, can be helpful in order to develop a common understanding of the situation and be consistent as a team. Always bring the nurse to the briefing and to the family meeting.	
Prognostication	
Prognostication involves assessing and communicating what to expect. Prognostic tools in severe brain injury patients have been developed, but are not reliable in individual patients. Individual assessments of prognosis must be made by the interdisciplinary team and must be based on all relevant medical information, both anamnestic, clinical, treatment response. Prognosis in the early stage after a head injury is difficult. Remember reassessments must occur when the condition is more clarified. What are the treatment goals? Are treatment goals realistic? Create a plan for re-evaluation; either time-based or milestone-based. In the most serious injuries, multiple complications may arise, recovery trajectories are long, possible recovery may come late. Cases of persistent disorder of consciousness are rare.	
Ethics	
Withdrawal of life-sustaining treatment should only occur after thorough interdisciplinary discussion (preferably during daytime). On duty, preliminary decisions about limitations should be made collaboratively by the anesthetist/ intensivist, the neurosurgeon and the surgical trauma-team leader. See the National ethics guidelines: “*Decisions should be based on what is reasonable from a medical and health-related point of view, what is in patient’s best interests and in line with what the patient wants. If there is doubt or uncertainty treatment should be started. Treatment should be continued until its benefit is better clarified. If there is doubt about benefit, the relatives should be informed that treatment might be withdrawn at a later point.*” The recognition that treatment is futile or potentially inappropriate may come already in the first evaluation after admittance or become evident later on. When treatment is recognized to be futile and total cessation of intracranial circulation can be expected, the team is obliged to think about donor detection.	

We interviewed senior consultants from OUH with extensive trauma care experience who were actively engaged in patient care. At OUH, end-of-life decisions are always made by, or at least run through senior consultants. We recruited participants by e-mailing all senior consultants via the heads of relevant departments, as well as by individual invitations. Our intention with the recruitment strategy was to offer the possibility to participate to all, but we nevertheless were eager to recruit the most engaged and experienced ones. E-mails provided background information and illustrations to prepare participants for the interviews. Participants were provided the Bosslet definitions of futility and potentially inappropriate treatment. Semi-structured interviews were conducted with 18 physicians who were affiliated with the neurocritical care unit: 9 neurosurgeons (N), 7 intensive care physicians (I) and 2 rehabilitation physicians (R). We included all the groups of involved specialities in neurocritical care to maximize the sample diversity. Among the 18 interviewed physicians were 7 women and 11 men. Their mean age was 53 years (range 38–73) and mean length of experience dealing with sTBI patients was 14 years (range 6–30). After 18 interviews saturation was obtained, which means further data collection cease to add understanding in relation to what has already been gained.

### The interviews

The interviews took place between April and September 2017, were conducted during regular working hours and lasted approximately 1 h each. The physicians were allowed by their head of department to liberate themselves from clinical task during this hour. Interviews were conducted, audiotaped and transcribed verbatim by AR. An interview guide was developed based on the researches expertise, relevant literature, the conceptual framework referred to in the introduction by Bosslet et al. and unresolved questions in a prior study conducted by our group on treatment-limiting decisions in sTBI in the trauma hospital [1, 8]. The unresolved question was if and how value considerations had impact on life-death decisions in the trauma hospital phase. The interview guide consisted of so called items to be covered, see Table 2. Using a list of items instead of fixed questions enables the researcher to use the guide in a flexible manner during the interviews. The guide, as is in-line with qualitative methodology, was slightly adjusted along the course of the study. In the interviews we wanted to *focus on the perceived “hard cases”*. Cases where there is a choice, a dilemma, where different considerations have to be balanced. According to common understanding in qualitative methodology the data is co-created by the interviewer and the participants [21]. The opening question in the interviews used to “set the stage” was: “Please share your experiences and reflections regarding encounters with severely brain injured patients and their families, when you were in doubt about whether to start, continue, withhold or withdraw life-sustaining treatment. Please use real-life cases you have been involved in. How did you perceive the situation at hand, how did you reason and act?” Open-ended questions were used to access descriptions in the physicians’ own words. Probing was used to further explore the meaning of what was said.

**Table 2:** Interview guide

How to conduct the interviews, examples	
Opening question to set the stage, see text in method section	
Ask the physicians to talk in a manner understandable for an outsider	
Probing: Please elaborate. Please clarify	
Items to be covered	
Strategies you use to deal with uncertainty and doubt:	
Crucial steps in the decision-making process	
Roles / collaboration within the teams	
To estimate and communicate prognosis	
Weight you give different considerations	
Patient’s values, wishes and will	
Impact of family input	
Timing issues; early vs late withdrawals	
Flipping roles: If you were the patient	
Concerns about current practice	
Additional comments: Please share	

### Analysis

Qualitative thematic analysis was used with the following analytic steps [22, 23]: 1. Reading of interviews for overall impression and searching for preliminary themes (AR, RF); 2. Re-reading, searching for meaning-units and coding of interviews word by word using inductive coding (AR); 3. Looking for similarities and differences across interviews, and nuances within interviews (AR, RF); 4. To understand the content in more depth the researchers developed the analysis further with a focus on two overarching questions; *why/in what kind of circumstances were physicians in doubt and how did they cope in situations when doubt was prevailing* (AR, RF, EH). In the analytic phase the research team moved back and forth between a position with critical thinking and interpretation of details such as single words or expressions to a bird view and search for the essence of what can be found in the text in relation to the research questions. The analysis was deliberately not constrained by any theory anchoring. NVivo Pro 11 (QSR International, Melbourne, Australia) was used as a tool to organize data and support our analysis [24]. To increase the quality of the analytic process, participants with different professional backgrounds were recruited: Two of the authors (AR and EH) were involved in clinical tasks related to some of the cases referred to; RF is a physician and an ethicist without clinical obligations and JHL is an intensivist without links to trauma care.

## Results

All physicians expressed that they had frequent feelings of doubt at some point during the decision-making processes. Doubt is not only rooted in diagnostic and prognostic uncertainty but also in values, experiences and emotions linked to each individual physician.

“It is seldom easy. When is there no doubt? When there is cessation of cerebral blood flow (brain death), but in all other cases, doubt is inevitable. Even though I have worked in the field for a long time, I feel humble and I am afraid to err.” 15_I

“In the admittance situation, I am regularly in doubt. I am well aware that my decision is a strong determinant of patients’ possibilities to survive and their possible outcome.” 3_N

The strongest doubt was in the admission setting, linked to lack of or conflicting information and to an awareness of a fallible clinical judgement in these early circumstances.

Doubt was also linked to possible interventions, how aggressively to extend them and at what costs for the individual. Closely linked to this is the question of what constitutes an acceptable quality of life (QoL) for this particular person. Physicians were vague about what they understood as an acceptable QoL, but they clearly expressed that the best possible functional outcome and an acceptable QoL were their overall long-term goals of treatment, not survival per se. Many of the physicians were humble recognizing that their own opinion about QoL may not be shared by their patients.

### Strategies applied by physicians to address uncertainty and doubt

#### Treatment trials

When in doubt, treatment trials or open-ended treatment would be attempted to give life a chance, even in situations where chances of a good outcome were extremely low.

“My starting point is the question: What can I do? Is there anything I can do? Then, usually, even in cases where I do not truly believe patients have a chance of a good outcome or even survival, I choose to start, I choose to give it a try ”11_N

Treatment that favoured life was especially prevalent when the patient was young.

“In young patients, I operate, and I start regardless of the prognosis. I want to try. With regard to older patients, I am more often in doubt. I tend to start even then, but I ask myself: What do I think I can achieve by treatment? What will I bring the patient back to?” 6_N

### Taking time as a coping strategy

When treatment is provided, time is also bought, and this is of paramount importance to follow individual treatment responses and allow for repeated clinical evaluations and reconsiderations.

“Following admission, within a short timeframe, the immediate injury dynamic is exposed. When we give situations a little time, we can often distinguish cases where we need to continue from cases where we feel confident that the right thing is to withdraw.” 7_I

### Collegial counselling and consensus seeking

Starting early on, the clinicians were engaged in interdisciplinary discussions and processes striving to access a set of different perspectives in which critical or questioning points of views from colleagues were welcomed.

“To know that different colleagues have somewhat different perspectives is enriching. Our meetings would be worthless if we all agreed about everything. I find it very valuable to be challenged in my way of thinking. After our discussions, I ask: Should I change my opinion, or do I still feel confident that my decision is ok?” 10_N

The interviews revealed that also when there could be diversity of opinions there was a common understanding that the physician closest to the patient had to be the one with the final say in difficult decisions but preferably after an open collegial discussion.

The collegial discussions helped physicians cope with stressful/negative emotions in circumstances of uncertainty. The support from colleagues and other team members, the broader trauma hospital discussion climate and the trauma hospital organization were of great importance. They appreciated what they perceived as a constant-learner culture in which critique is necessary but also accepted.

“It was during our morning discussions with all the neurosurgeons present. The team on-call had operated on a truly awful injury during the night. We heard the story and examined the CT scans. I thought, I would not have done it. One of the seniors said: This was truly a heroic effort. I thought it was such a nice way to comment. He did not criticize, but as I interpreted it, he told them that what they had done was truly *above and beyond* what could reasonably be expected of them*,* and it would have been perfectly all right to have said no.” 3_N

The open collegial discussions often led to a need to adjust the judgement and the plan. Thus, adaptive approaches were described as key to dealing with uncertainty; take one-step back, re-evaluate, and sometimes allow for surprising turn-arounds.

“In daylight things look different. The team on duty had decided not to operate on a devastating case, but the next morning, we changed our mind. From time to time, we change our mind without having new information because we think differently when we apply collective thinking, looking at the situation with new eyes.” 9_N

### Medical framing

The physicians revealed that they primarily focused on the medical considerations. The wishes and will of the patient was absent from their early decision-making. Some completely rejected the value of preinjury statements such as advance directives or even questioned the utility of the concepts such as ‘patient’s best interest’ or ‘patient’s wishes’.

“An advance directive? No, in my opinion it would be useless. I would not have looked at it. The medical situation is what carries weight in this context, not the patient’s wishes.” 4_N

### Variability between individual physicians

The degree of doubt and how they dealt with it varied between individual physicians, also within one specialty. A few stated that they should not let their doubt dominate. They focused on their professional responsibility and how to apply their best possible situational judgement purely built on the available medical knowledge, their clinical neurological examination, CT scans and overall medical judgement.

Others felt very humble and afraid to err because they had experienced how their best professional judgement had been wrong.

The interviews revealed great variability in the physicians’ descriptions of factors influencing their decisions, such as intuition and feelings.

“The more medical uncertainty there is, the more I must rely on my intuition, the X factor” 3_N

Some described how their own feelings, especially in cases with small children, influenced their judgement and how they sometimes identified with patients or families to a degree that threatened their objectivity. In lacking knowledge about the patient, they described how they turned to thoughts of what they would have wanted for themselves, their children or for a parent.

What kind of treatment they would have wanted for themselves if they had been in a situation of devastating brain injury varied:

“I must admit my tolerance for the thought of surviving with severe neurological damages is low. I would have preferred to die. However, I think I am able to separate these things; what I want for myself and the value-thinking I apply when I make decisions for others” 7_I

They also described how they sometimes felt at risk of being biased by cases they themselves had been exposed to, particularly cases with unexpected outcomes. Some described how they often were deeply touched by and stored in their mind the cases with exceptional good recovery, as well as cases they had been involved in that never recovered as hoped for.

“He was admitted with a devastating brain injury with absolutely all signs of “do not touch”, all the traditional bad prognostic signs, but somehow I felt I should operate on him. He survived and made an exceptional recovery. These are the outliers that “*bug*” our decision-making.” 10_N

In addition to variability in descriptions about factors influencing decisions, they also described how treatment intensity varied among their colleagues. This was perceived as being in part tied to their personality and temperament and in part to their level of experience.

“Some surgeons are very aggressive. They are always pursuing the goal to rescue life. Others more often say; this will not work. It is linked to personality.” 9_N

“Some neurosurgeons are less experienced and may err in early judgements. The experienced neurosurgeons may also err, but probably less often. I have been told that you need 5 years to learn to operate but 15 years to learn when not to operate. Therefore, yes, there is doubt, and I am afraid to err.” 6_N

## Discussion

The context of this study is the trauma hospital and the first days of neurocritical care for patients with devastating brain injuries. Following severe brain injury, decision-makers are faced with multilayer uncertainty [25]. How physicians deal with uncertainty is one of the core issues in neurocritical care [26]. Our study indicates that the practice of neurocritical care physicians is largely in accordance with national and international guidelines. However, guidelines do not eliminate a need for discernment, and accordingly doubt in individual cases.

Doubt has cognitive and emotional aspects. The cognitive aspect relates to uncertainties in assessing the benefits and the harm of aggressive care and may be due to incomplete clinical information or to a lack of precision of clinical prediction models. The emotional part relates to the feelings that arise in decision-makers when confronted with a difficult choice. This amounts to moral ambiguity; what is the *right* decision, i.e., the one that best balances risks and benefits with patients’ values and preferences?

### Ethical concerns linked to doubt in early decision-making after devastating brain injury

#### Doubt and variability, but aiming for consensus

Variations in end-of-life practices have been found between countries, regions, physicians from different specialities and between individual physicians [4, 15, 27]. Our study confirms that there is interphysician variability regarding doubt and how doubt is dealt with within a single institution.

Variability can be thought of as a matter of concern, potentially leading to unequal or suboptimal care. On the other hand, variability is inevitable because it reflects human diversity and physicians´ flexibility, and attention and sensitivity to subtle and unmeasurable aspects of critical illness and dying [28]*.* Interestingly, the physicians in our study expressed an acceptance of the variability they witnessed, both in practice and in their colleagues’ attitudes. They described how they strove to reduce variability by being constant learners, being open to input from others and open to think “maybe I am the one that got it wrong, or maybe my judgement is somehow biased” [29]. From a starting point of doubt and different opinions, they tried to move towards consensus whenever possible.

### Doubt about benefits and harms

Feelings of doubt may serve as a stop sign, to clarify what is needed in order to obtain a strengthened decision-making position.

Physicians in our study describe their aggressive early interventions as medical necessities that were rooted in their obligation to provide treatment in emergency situations and undertaken in patients’ best interest. In accordance with guidelines, aggressive treatment is provided even if individual physicians have little or no faith in its usefulness. This may be conceptualized as overtreatment, but it can also be understood as a safeguard against irreversible decisions made too early. In these circumstances, other ethical considerations, such as patients’ values and preferences, are suppressed.

### Undue influences of physicians’ values, emotions and cognitive bias

Our study indicates that a doctor’s emotions may influence end-of-life decision-making [30]. Some may consider doubt as a threat to physicians´ authority, which may reduce families’ and patients’ trust in physicians. Honesty about doubt and humility may also be seen as true signs of excellence [31].

Value issues, such as quality of life prognostications, may be part of their bias in determining the best solution for individual patients. The underlying assumptions behind these value-based considerations are rarely made explicit. Implicit values may be a threat to high-quality decision-making and are in contrast to a culture built on transparency and ethical deliberation.

Our study also reveals that physicians can reflect on how their own emotions and cognitive biases may impact decision-making for their patients, but emotions and biases are not often discussed openly among colleagues. Undue influence of physicians’ emotions and values requires specific attention [14].

Another issue is the conscious or subconscious risk of amplification of shared values as a result of group dynamics within a hospital subculture. Consensus in an interdisciplinary team is no guarantee of good decision-making if the goals for good decisions are to be both reasonable from a medical point of view and patient-tailored [32, 33].

### Medical framing and the absence of the patient’s voice

Our physicians reveal that early decisions are made without regard to patients’ values or preferences. This may be criticized. There may be situations where a patient has made explicit decisions about future life-prolonging treatment. If clinicians always exclude information about patient values in their early decisions, when do they start seeing this information as relevant? It is unclear whether families of patients with poor prognoses who receive aggressive treatment are provided with opportunities to express their views of what their close relatives would have wanted. This represents an ethical concern.

### How to move forward?

To foster good decision-making, the ethics climate of an institution is as important as medical expertise, adherence to guidelines and system factors. The question is whether it is given enough attention in trauma hospital settings or whether it needs to be further developed?

A good ethics climate is built on a culture of ethical awareness and open interdisciplinary reflection [34]. Physicians need to be self-reflective about their role as decision-makers [14, 35]. Acceptance of doubt is, as we see it, an essential part of a good ethics climate and is necessary for high-quality care.

Acknowledgement of doubt can protect against premature decision-making and can cause receptiveness to the perspectives of colleagues, nurses, families and patients. Acknowledgement of doubt can also be used to clarify dilemmas. If a physician manages to be explicit about when and why he or she is in doubt, the situational understanding among the other team members and family may improve. Unrealistic expectations can be avoided, e.g., in circumstances of early aggressive treatment. Sharing doubt can help families maintain hope with a more nuanced and realistic understanding of the range of possible outcomes.

Although doubt is an inescapable part of neurocritical care, it has not been studied systematically and transparently. Understanding the implicit values and preferences that influence physicians’ thinking may teach us more about how to find solutions when ethical dilemmas arise and may help us to identify areas where improvement is needed.

### Norms are moving

Benoit et al. describe a set of crucial elements in developing a good ethics climate within an institution [34, 36]. One of them is the importance of physicians’ self-reflections and self-awareness. Emotions and attitudes are understood as important elements in shaping decisions and should be shared openly. Different opinions and values towards end-of-life issues must be expected and should be tolerated. Physicians in charge should help team members settle their differences.

The norm of consensus is challenged by Wilkinson et al. [33]. They claim that value pluralism and moral uncertainties in end-of-life means that it is unrealistic and sometimes counterproductive to seek unanimous or even majority level agreement. A process of reasoned discussions, elucidation of facts and exploration of values are worthwhile even if agreement is not forthcoming. Not one, but a range of reasonable decisions may be acceptable. Professionals should agree to respect views that they do not personally share.

Laurent et al. claim end-of-life decisions frequently evoke strong feelings among heath care personal, nevertheless professionals seem to have a poor understanding or often overlook the emotional dimensions of their work [30]. They suggest to share and validate emotional dimensions, and allow them to be viewed as resources that shield light on end-of-life decisions in the intensive care units.

#### Limitations and strengths of the study

In this study, physicians were interviewed. Nurse, family and patient perspectives are also needed to obtain a richer and more complete picture of decision-making in the neurocritical care context. We have a narrow focus on cases of potentially inappropriate treatment and on physicians’ doubt. The primary investigator knew the interviewed physicians personally. She was familiar with some of the cases as well as the hospital culture through her clinical work as an intensivist. Some issues may be easier to address with a total stranger, but we believe the familiarity between the participant and interviewer fostered honest and open reflections. However, closeness to the research topic can produce bias; therefore, the last author, who is not employed in clinical practice, but is an ethicist familiar with the value issues in end-of-life decision-making actively participated in all steps throughout the research process in particular the development of the interview guide and the interpretation of all interviews. One strength, but also a problem, that arises when the interviewer and the interviewed share the same subculture is that there exists an implicit common ground underlying their conversation, with risk of creating blind spots.

## Conclusion

A need for clarity must be reconciled with an acceptance of residual uncertainty to enable high quality real-life decisions in DBI. Accordingly we believe that the ability to embrace doubt as part of the process is important to improve the quality of decision-making processes and that this warrants more attention. Doubt is linked to the recognition of the fallibility of medical judgements and can be seen as an important source of open-mindedness. Negative emotions that may arise from doubt are mitigated by open discussions that acknowledge doubt as a rational and normal feature of complex decision-making.

## Data Availability

The approval from the Institutional Data Protection Officer did not include free sharing of data.

## Abbreviations

DBI: Devastating brain injury
OUH: Oslo University Hospital
PIT: Potentially inappropriate treatment
QoL: Quality of life
sTBI: Severe traumatic brain injury
TLD: Treatment-limiting decisions
